# Association between time-weighted average lactate during cardiopulmonary bypass and postoperative infection in patients undergoing cardiac surgery: single-center, prospective cohort study protocol

**DOI:** 10.3389/fcvm.2026.1830791

**Published:** 2026-08-12

**Authors:** Yiwei Lin, Shujie Wang, Yaqi Han, Jingyu Li, Wei Feng, Zhichao Li, Youzhuang Zhu, Yang Zhao

**Affiliations:** 1 Department of Anesthesiology, The Affiliated Hospital of Qingdao University, Qingdao, Shandong, China; 2 Department of Anesthesiology, National Cancer Center/National Clinical Research Center for Cancer/Cancer Hospital, Chinese Academy of Medical Sciences and Peking Union Medical College, Beijing, China

**Keywords:** cardiac surgery, cardiopulmonary bypass, infectious diseases, lactate, sepsis

## Abstract

**Introduction:**

During cardiopulmonary bypass (CPB), impaired perfusion, ischemia–reperfusion injury, and systemic inflammation frequently result in lactate accumulation. Lactate is routinely monitored perioperatively and may serve as an early indicator of postoperative infection, yet prospective evidence linking intraoperative lactate to infection risk remains limited. This prospective cohort study aims to evaluate the association between the time-weighted average (TWA) lactate during CPB and the risk of postoperative infection in patients undergoing cardiac surgery.

**Methods:**

This single-center, prospective cohort study will enroll 346 adult patients undergoing elective cardiac surgery with CPB. Arterial blood samples will be obtained at multiple intraoperative time points to calculate TWA lactate. Patients will be classified into exposure and non-exposure groups based on a TWA lactate cutoff of ≥2.0 mmol/L. The primary outcome is the incidence of infection within 30 days of surgery. A predefined directed acyclic graph will guide covariate adjustment. Multivariable logistic regression will estimate the risk association, and restricted cubic spline models will explore potential nonlinear relationships. Sensitivity analyses will use lactate load, defined as the area under the lactate–time curve during CPB, to examine whether cumulative intraoperative lactate exposure yields results consistent with those obtained using TWA.

**Discussion:**

This prospective cohort study aims to investigate the association between time-weighted average lactate during cardiopulmonary bypass and postoperative infection risk in cardiac surgery patients. If the hypothesis is confirmed, TWA lactate could serve as a readily available marker for intraoperative risk stratification, facilitating early identification of high-risk patients and guiding individualized interventions. The findings may provide evidence for optimizing perioperative management and improving clinical outcomes. As a single-center study, external validation in multicenter settings will be needed to enhance generalizability.

**Clinical Trial Registration:**

https://www.chictr.org.cn, identifier ChiCTR2500110269.

## 1 Introduction

Cardiovascular disease remains the leading cause of death and disability worldwide, causing 19.2 million deaths globally in 2023 (1). Cardiopulmonary bypass (CPB)-assisted cardiac surgery is a common and effective clinical intervention (2, 3); however, the pathophysiological alterations induced by CPB markedly increase the incidence of perioperative complications, particularly postoperative infections (4). The major types of postoperative infections include sepsis, pulmonary infection, surgical site infection, mediastinitis, and infective endocarditis (5). These complications have contributed to increased healthcare costs, prolonged hospitalization, and poorer patient outcomes. Therefore, identifying early and measurable risk markers is of great clinical significance in reducing postoperative infections, mortality and healthcare resource utilization.

**Figure 1:**
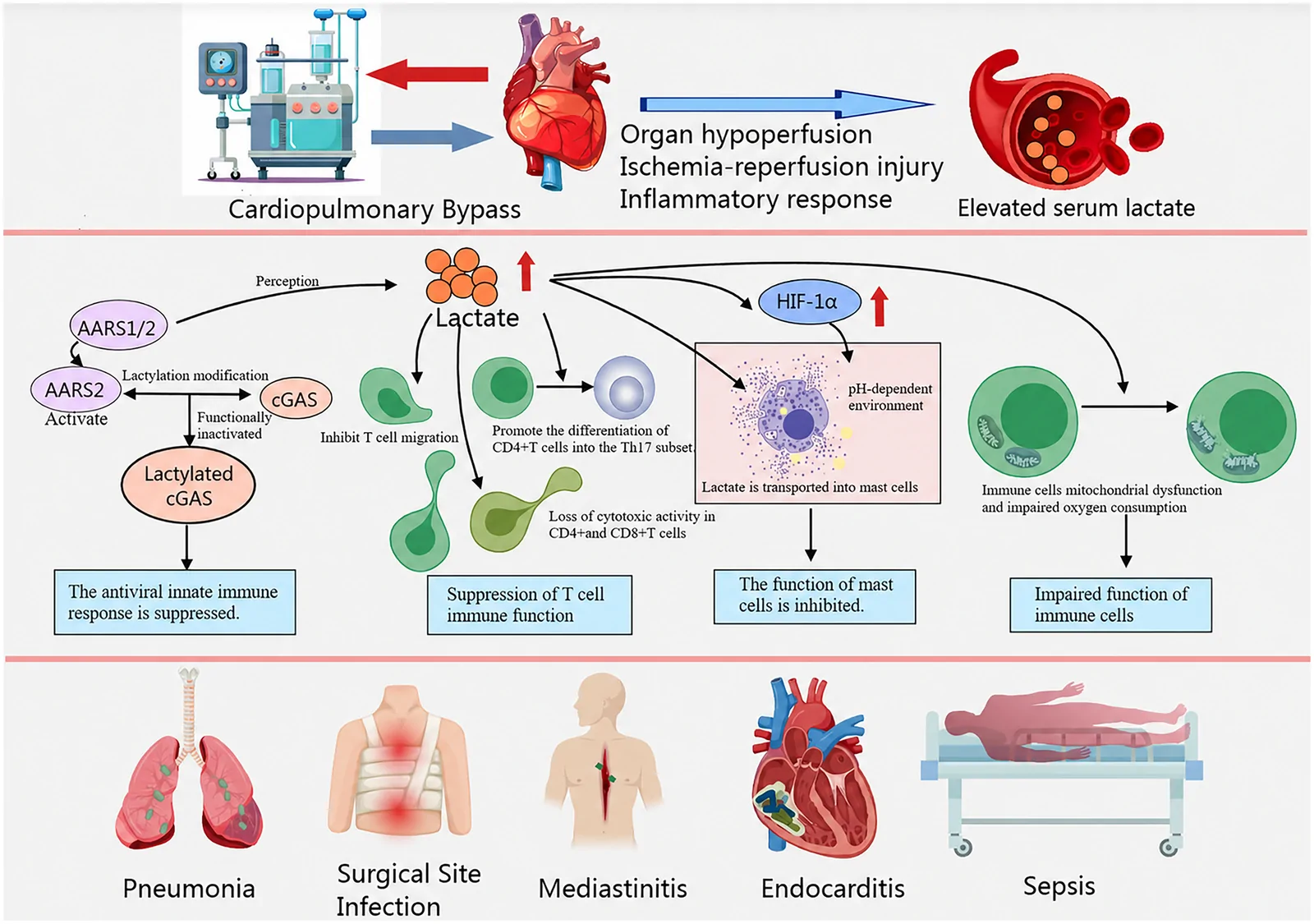
Proposed mechanism by which TWA lactate during cardiopulmonary bypass induces immunosuppression leading to postoperative infection. During cardiopulmonary bypass, non-pulsatile perfusion, organ hypoperfusion, ischemia–reperfusion injury, and systemic inflammatory activation may promote serum lactate accumulation. Elevated lactate may subsequently contribute to perioperative immune dysregulation through several mechanisms, including AARS1/2-mediated lactate sensing and cGAS lactylation, suppression of T-cell migration and cytotoxic function, promotion of CD4⁺ T-cell differentiation toward the Th17 subset, impairment of mast-cell function in a pH-dependent microenvironment, and mitochondrial dysfunction with reduced oxygen consumption in immune cells. Collectively, these lactate-related immunosuppressive effects may create a favorable environment for pathogen colonization and dissemination, thereby increasing the risk of postoperative infectious complications, including pneumonia, surgical site infection, mediastinitis, infective endocarditis, and sepsis. TWA, time-weighted average; CPB, cardiopulmonary bypass; AARS1/2, alanyl-tRNA synthetase 1/2; CD4⁺ T-cell, cluster of differentiation 4-positive T cell; CD8⁺ T-cell, cluster of differentiation 8-positive T cell; cGAS, cyclic GMP-AMP synthase; HIF-1*α*, hypoxia-inducible factor-1*α*; Th17, T helper 17 cell.

Serum lactate is a complex biomarker. Lactate levels during CPB surgery typically serve as an indicator of overall physiological stress, surgical complexity, and perfusion adequacy (6, 7). The nonpulsatile perfusion pattern during CPB can lead to organ hypoperfusion, ischemia–reperfusion injury, and systemic inflammatory responses, all of which may enhance lactate metabolism and cause peripheral lactate accumulation (8, 9). In this study, we hypothesize that lactate serves as both a marker of underlying pathophysiological processes and a potential mediator of immunosuppression. At high concentrations (e.g., 12.5 mM), it enhances hypoxia-inducible factor 1-alpha (HIF-1*α*) expression, while mitigating acute inflammatory injury, which also suppresses mast cell activation via monocarboxylate transporter 1 (MCT-1) and pH-dependent pathways, thereby increasing the risk of infection (10). Furthermore, lactate impairs T-cell function by inhibiting migration, reducing the cytotoxic activity of neutrophil, cluster of differentiation 4-positive T cells (CD4^+^) and cluster of differentiation 8-positive T cells (CD8^+^) T cells, promoting CD4^+^ T cell differentiation toward the T helper 17 cells (Th17) subset, and inducing interleukin-17 (IL-17) accumulation, collectively leading to the occurrence of surgical site infections and other infections (11, 12). In sepsis, lactate is closely associated with diagnosis, progression, and treatment, and it further reduces immune cell activity by inducing mitochondrial dysfunction and impaired oxygen consumption, contributing to multiple organ dysfunction (13). Lactate can also suppress nuclear factor *κ*B (NF-*κ*B) transcriptional activity and cytokine production in mast cells, whereas enhancing glycolysis and adenosine triphosphate (ATP) generation can restore immune function (14). Previous studies have also indicated that acidification is an independent virulence mechanism for pneumonia barrier disruption and inflammatory dysregulation, which may directly contribute to the development and progression of bacterial pneumonia, whereas maintaining normal pH represents an effective therapeutic strategy for preserving barrier integrity and immune response (15). Based on the above evidence, a mechanistic diagram (Figure 1) was constructed to integrate these proposed mechanisms and illustrate how cardiopulmonary bypass-related lactate elevation may promote postoperative infection through lactate-mediated immune dysregulation. Because lactate measurement is inexpensive, reproducible, and routinely monitored during the perioperative period, it has inherent advantages as a potential biomarker for postoperative infection risk.

Currently, studies on the association between lactate levels and the risk of postoperative infection in cardiac surgery are limited and the evidence is insufficient. Studies on lactate and cardiac surgical outcomes have primarily focused on organ dysfunction, mortality, intensive care unit (ICU) length of stay, and postoperative mechanical ventilation time (16, 17). In the field of infection research, investigations of lactate have mainly centered on its role in assessing sepsis severity and prognosis (18). Moreover, a retrospective study of adult cardiac surgery found that there was no significant difference in the incidence of postoperative infection between the high lactate group and the low lactate group (19). However, another more recent retrospective study found that patients with postoperative pneumonia had significantly higher perioperative lactate levels than those without pneumonia (20). Therefore, in this particular population of cardiovascular surgery patients, the lactate levels during surgery and the incidence of various types of postoperative infections, such as pulmonary infection, surgical site or mediastinal infection, and infective endocarditis, still lack large-scale prospective cohort studies.

In patients undergoing cardiac surgery with CPB, evaluating the predictive value of lactate exposure during CPB as an early warning indicator for postoperative infection may help address current evidence gaps. It can provide a scientific basis for perioperative management, early infection identification, and individualized prevention strategies, thereby reducing complication rates and healthcare burdens. Therefore, the relationship and predictive value between lactate and postoperative infection warrant validation through larger, prospective cohort studies, underscoring the clear clinical and scientific significance of the present research.

This study is a single-center, prospective cohort study designed to investigate the association between time-weighted average (TWA) lactate levels during CPB and postoperative infection in patients undergoing cardiac surgery. The null hypothesis of this study is that the time-weighted average lactate level during CPB is not associated with postoperative infection in patients undergoing cardiac surgery with CPB.

## 2 Methods

### 2.1 Study design and setting

**Figure 2:**
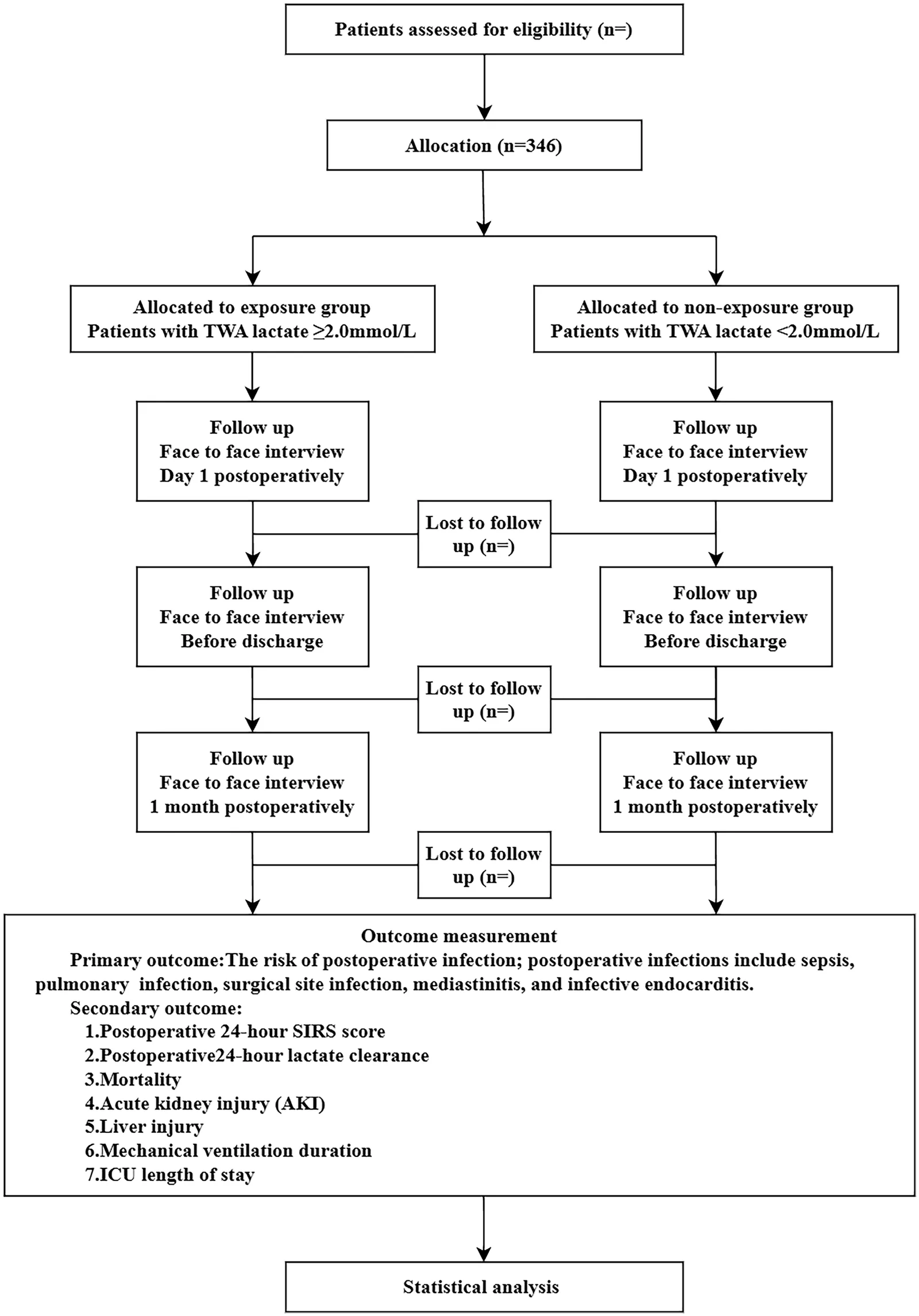
Study flow diagram.

This study is a single-center, prospective cohort study. It is planned to be conducted at the Affiliated Hospital of Qingdao University. Participants will be categorized into an exposure group based on a TWA lactate level ≥2.0 mmol/L and followed for 30 days postoperatively to assess the occurrence rate of postoperative infections (Figure 2). This trial was approved by the Medical Ethics Committee of the Affiliated Hospital of Qingdao University (Approval No. QYFYEC2025-147). Prior to study initiation, written informed consent will be obtained from all participants. This study will be conducted in accordance with the Declaration of Helsinki. The trial has been prospectively registered with the Chinese Clinical Trial Registry (ChiCTR2500110269, available at https://www.chictr.org.cn, prospectively registered on October 11, 2025).

### 2.2 Eligibility criteria

#### 2.2.1 Inclusion criteria

1. Age ≥18 years; 2. Patients undergoing elective open cardiac surgery with CPB, such as coronary artery bypass grafting, valve replacement or repair, artificial vascular replacement, cardiac tumor resection, other, and combined surgery, such as valve replacement combined with coronary artery bypass grafting.

#### 2.2.2 Exclusion criteria

1. History of previous cardiac surgery requiring sternotomy and/or CPB; 2. Emergency surgery; 3. Presence of any type of preoperative infection (e.g., preoperative pneumonia, infective endocarditis); 4. Severe hepatic or renal dysfunction (e.g., jaundice, coagulation disorders, hepatic encephalopathy, serum creatinine >442 μmol/L, blood urea nitrogen >20 mmol/L, or requiring dialysis); 5. Preexisting immunodeficiency disorders (e.g., HIV infection, use of immunosuppressive drugs, autoimmune diseases).

### 2.3 Sample size calculation

Previous studies have reported that the incidence of postoperative infection after cardiovascular surgery is approximately 10.4%–15.5% (21–23). To obtain a conservative sample size estimate and avoid overestimating the expected event rate, we used the lowest reported incidence of 10.4% as the baseline infection rate in the normal lactate group. The same study that reported this baseline incidence also reported an odds ratio (OR) of 2.75 for postoperative infection in the high-lactate group compared with the normal-lactate group (21). With a two-sided *α* of 0.05 and a power of 0.9, the required sample size per group was estimated to be 156 using PASS 2021 software (NCSS, LLC, Kaysville, UT, USA), resulting in a total sample size of 312. Considering a 10% loss to follow-up, at least 173 participants per group are needed, for a total of 346 participants.

### 2.4 Exposure

Trained research personnel will measure patients' blood lactate levels during cardiopulmonary bypass using Radiometer ABL90 FLEX blood gas analyzer. The procedures are as follows:

1. During cardiopulmonary bypass, arterial blood samples will be collected from the radial or femoral artery at the following time points: T_1_, after the initiation of cardiopulmonary bypass, when coronary perfusion with cardioplegic solution induces cardiac arrest; T_2_, T_3_, …, Tn, every 1 h after the start of bypass; T_n_ _+_ _1_, 10–20 min before the end of bypass; and T_n_ _+_ _2_, within 5 min after the end of bypass. Here, T_n_ represents the last full-hour sampling point during bypass. Additionally, extra samples will be collected whenever there is a sudden increase in lactate during CPB (e.g., following hypotension or hypoperfusion events). At least four lactate measurements during cardiopulmonary bypass are required for TWA lactate calculation.
2. Within 1 min of blood collection, lactate levels will be measured using the blood gas analyzer and recorded in mmol/L to one decimal place. If duplicate measurements are performed at the same time point, the mean value will be used as the final lactate measurement.
3. The time-weighted average lactate (TWA) will be calculated from the lactate values obtained at different time points. TWA is defined as the area under the curve of lactate levels over time divided by the total observation interval, using the formula:
  $$TWA=\frac{1}{T}\overset{n=1}{\underset{i=1}{\sum }}\;\left(\frac{v_{i}+v_{i+1}}{2}\right)\Delta t_{i}$$
4. Evidence from previous cardiac surgery cohort studies has reported that lactate concentration of >2.0 mmol/L during surgery or in the early postoperative period are already associated with increased postoperative morbidity and mortality. Accordingly, based on the available evidence, recommendations from international sepsis guidelines, TWA lactate of 2.0 mmol/L is selected as the prespecified cutoff for exposure classification. TWA lactate ≥2.0 mmol/L during CPB will be categorized into the exposure group, with the TWA lactate <2.0 mmol/L classified into the non-exposure group (24–27). Lactate will also be analyzed as a continuous variable in the subsequent analyses.

We will also calculate the following lactate indices to comprehensively characterize lactate exposure:

1. Peak lactate: The highest lactate value recorded during CPB.
2. Lactate load: The area under the lactate-time curve (AUC) during cardiopulmonary bypass, calculated using the trapezoidal method.
3. 24-hour postoperative lactate clearance: [(Lactate at the end of surgery – Lactate at 24 h after surgery)/Lactate at the end of surgery]  ×  100%.

### 2.5 Assessment of postoperative infection

Postoperative infections will be evaluated by an independent, professionally trained review panel, including attending or higher–level cardiac surgeons, ICU physicians, and infectious disease specialists. All adjudicators will remain blinded to patients' lactate exposure group assignment and TWA lactate values. Postoperative infections to be recorded include sepsis, pulmonary infection, surgical site infection, mediastinitis, and endocarditis. The frequency of postoperative infection assessment is shown in Table 1.

**Table 1:** Schedule of data collection and measurement time points.

Outcome measure Time		Visit 1	Visit 2	Visit 3	Visit 4	Visit 5	Visit 6	Visit 7	Visit 8
		Presurgery	During operation	Postoperative day 1	Postoperative day 2	Postoperative day 3	Postoperative day 7	Postoperative day 14	Postoperative day 30
Enrollment	Eligibility screening	×							
	Informed consent	×							
	Demographic characteristics	×							
	Baseline measures	×							
	Preoperative comorbidities	×							
Intraoperative	Surgery-related variables		×						
	physiological and anesthesia-related		×						
Postoperative	laboratory indices			×					
	SIRS score			×					
	GCS score			×	×	×	×	×	×
	Sequential Organ Failure Assessment Score			×	×	×	×	×	×
	Postoperative infection			×	×	×	×	×	×
	Acute kidney injury				×		×		
	Liver injury					×			
	ICU length of stay			×	×	×	×	×	×
	Mechanical ventilation duration			×	×	×	×	×	×
	Postoperative Adverse Events	Safety outcomes will be assessed from admission through 30 days post-surgery							

SIRS score, systemic inflammatory response syndrome score; GCS score, glasgow coma scale score.

#### 2.5.1 Postoperative pulmonary infection

It is defined as the presence of new or progressive infiltrates on chest radiography or CT within 30 days postoperatively, along with at least two of the following three criteria: (1) fever >38 °C without other causes; (2) white blood cell count >12  ×  10⁹/L or <4  ×  10⁹/L; (3) purulent secretions or positive sputum culture results (28).

#### 2.5.2 Sepsis (Sepsis-3)

It is defined as an acute change of ≥2 points in the Sequential Organ Failure Assessment (SOFA) score caused by infection within 30 days postoperatively. The SOFA score is a scoring system used to evaluate the severity of organ dysfunction in critically ill patients. It covers six major organ systems: respiratory, cardiovascular, hepatic, renal, neurological, and coagulation. Each organ system is scored from 0 to 4, with higher scores indicating more severe organ dysfunction (29–31).

#### 2.5.3 Surgical site infection

It is defined as the occurrence of at least one of the following in the surgical incision within 30 days postoperatively: (1) purulent drainage from the deep incision, not originating from the organ/space of the surgical site; (2) spontaneous dehiscence of the deep incision or surgical opening by a surgeon due to infection, accompanied by at least one of the following: fever (>38 °C), localized pain, or tenderness; (3) evidence of abscess or other deep incision infection detected by direct examination, reoperation, histopathology, or imaging; (4) diagnosis of deep incision infection by the surgeon or attending physician (32).

#### 2.5.4 Mediastinitis

It is defined as a wound infection involving the sternal bone within 30 days postoperatively, with or without involvement of the retrosternal space (33). Retrosternal involvement will be determined based on radiologic evidence (contrast-enhanced CT or MRI), supported by intraoperative findings or microbiological culture when available.

#### 2.5.5 Infective endocarditis

It is defined as meeting both of the following criteria within 30 days postoperatively: (1) positive blood cultures for common pathogens of infective endocarditis (such as staphylococcus aureus and streptococcus viridans); (2) echocardiographic evidence of infective endocarditis (34).

### 2.6 Covariates

**Figure 3:**
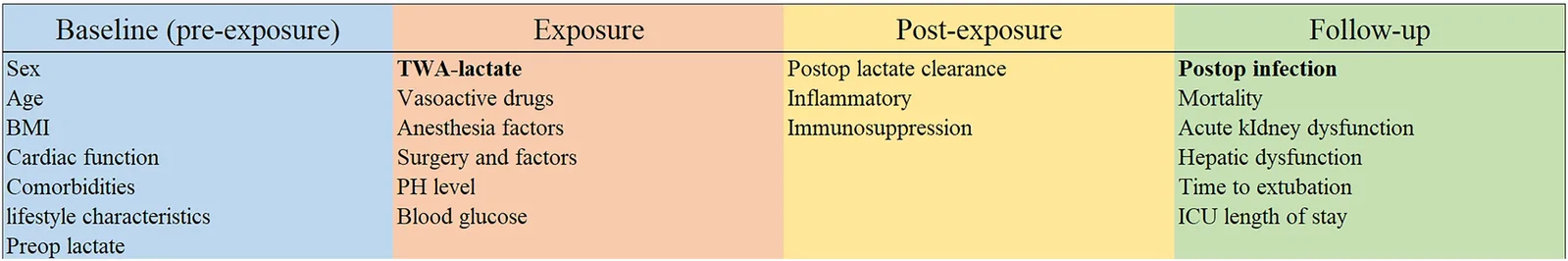
Temporal sequence of variables included in the causal framework.

**Figure 4:**
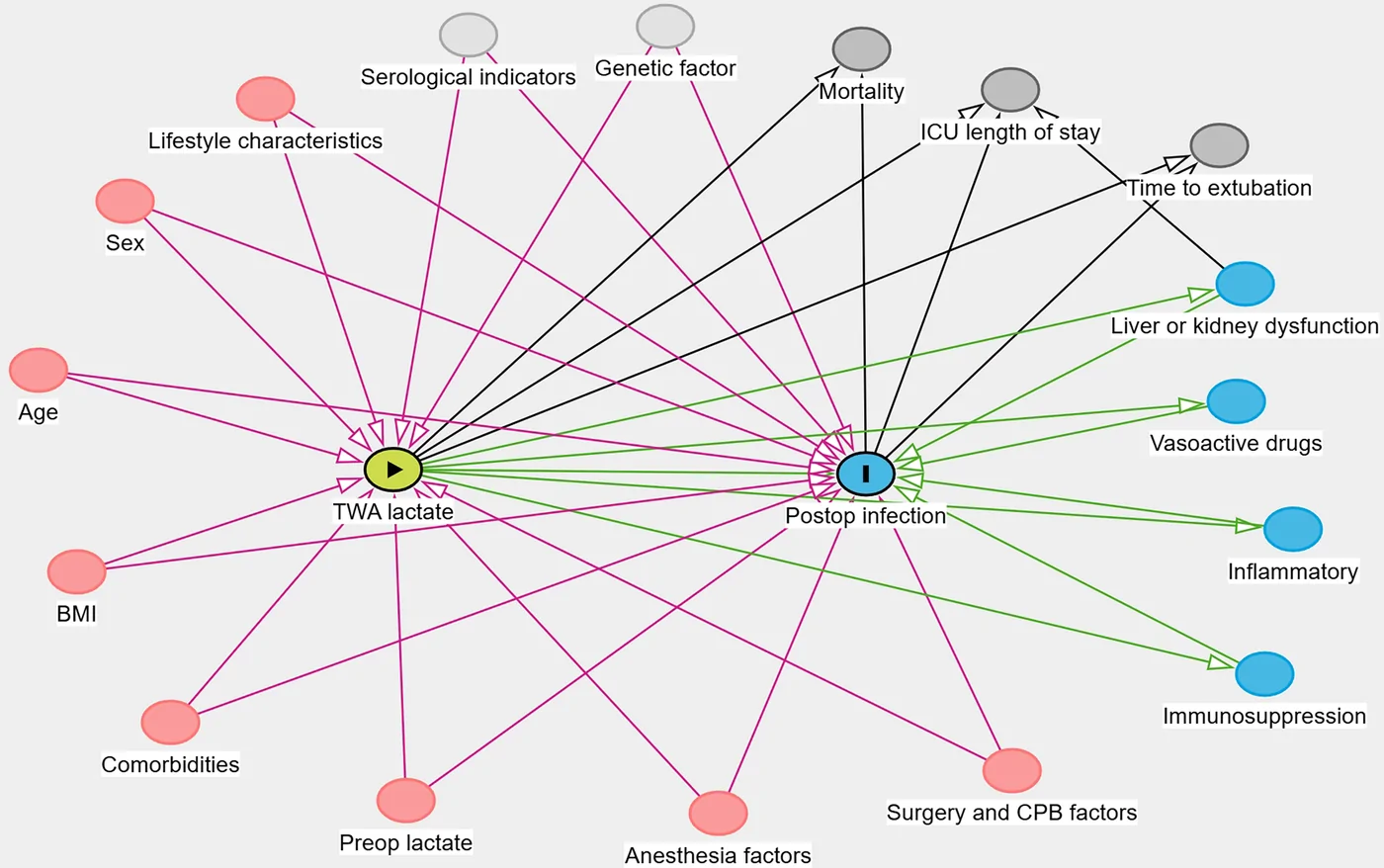
Directed acyclic graph (DAG) illustrating the assumed causal structure between intraoperative TWA lactate (exposure) and postoperative infection (outcome). The exposure is represented by a green node with a triangle symbol. The outcome, postoperative infection, is shown as a blue node with a thick black vertical line. Other variables are represented as follows: blue nodes depict ancestors of the outcome, namely mediators; red nodes represent ancestors of both the exposure and outcome; pale grey nodes signify unobserved or latent variables; and darker grey nodes represent colliders. The definitions of TWA lactate and postoperative infection have been elaborated in the Methods section. Mediators located on the causal pathway between exposure and outcome, as well as colliders jointly caused by both exposure and outcome, will not be adjusted for to avoid overadjustment bias. The minimal adjustment set for this model is age, sex, BMI, lifestyle, comorbidities, preoperative lactate, anesthesia factors, and surgery and CPB factors.

To ensure appropriate confounder control and strengthen inference, a directed acyclic graph (DAG) and temporal sequence diagrams for variables were constructed based on prior clinical knowledge and published evidence (Figures 3, 4). These diagrams clarify the assumed causal pathways linking intraoperative TWA lactate to postoperative infection. The DAG identified the following minimal sufficient adjustment set: age, sex, BMI, lifestyles (including smoking and alcohol consumption), comorbidities, preoperative lactate, anesthesia factors, surgery and CPB factors (including surgery type, operation duration, CPB time, aortic cross-clamp time, and intraoperative body temperature). These variables are common causes of elevated lactate during cardiopulmonary bypass and of postoperative infection risk. Mediators include hepatic and renal dysfunction, lactate clearance, inflammatory response, and immunosuppression. ICU length of stay, hospital length of stay, time to extubation, and mortality are considered collider variables.

#### 2.6.1 Demographics and baseline characteristics

Age (years), sex (male/female), BMI (kg/m^2^), smoking history (yes/no; defined as continuous or cumulative smoking for more than six months), alcohol consumption (yes/no; defined as drinking ≥1 time per month for ≥1 year), and NYHA functional class (I: patient has heart disease but no limitation of physical activity; ordinary physical activity does not cause undue fatigue, palpitations, dyspnea, or angina; II: slight limitation of physical activity due to heart disease; III: marked limitation of physical activity due to heart disease; IV: heart disease with symptoms of heart failure or angina at rest, any physical activity increases discomfort).

#### 2.6.2 Preoperative comorbidities

Hypertension (systolic BP ≥140 mmHg or diastolic BP ≥90 mmHg or taking antihypertensive drugs, yes/no), diabetes (fasting glucose ≥7.0 mmol/L, 2-hour postprandial glucose ≥11.1 mmol/L, random glucose ≥11.1 mmol/L, or taking antidiabetic drugs, yes/no), arrhythmias (including atrial arrhythmia, ventricular arrhythmia, atrioventricular block, yes/no), history of cerebrovascular disease (including cerebral infarction or hemorrhage, yes/no), hypoproteinemia (serum total protein <60 g/L or serum albumin <35 g/L, yes/no), pulmonary comorbidities (including chronic obstructive pulmonary disease, asthma, interstitial lung disease, pulmonary edema, yes/no), anemia (adult male <120 g/L, adult female <110 g/L, yes/no) and obesity (BMI ≥30 kg/m^2^, yes/no).

#### 2.6.3 Preoperative laboratory and functional indicators

Lactate level (mmol/L), platelet count (10⁹/L), hemoglobin (g/L), lymphocyte count (10⁹/L), neutrophil count (10⁹/L), monocyte count (10⁹/L), B-type natriuretic peptide (BNP, pg/mL), C-reactive protein (CRP, mg/L), left ventricular ejection fraction (LvEF, %), cardiac troponin I (CTnI, μg/L), myoglobin (Mb, μg/L), total bilirubin (*μ*mol/L), serum creatinine (μmol/L), alanine aminotransferase (U/L), aspartate aminotransferase (U/L), blood urea nitrogen (mmol/L), and preoperative blood glucose (mmol/L) within seven days before surgery.

#### 2.6.4 Surgical factors

Type of surgery (coronary artery bypass grafting, valve surgery, major vascular surgery, combined surgery and other), surgical duration (min), CPB time (min), aortic cross-clamp time (min), anesthesia duration (min), pH and blood glucose levels (mmol/L) from arterial blood gas analysis from the initiation of cardiopulmonary bypass to the end of surgery, body temperature before surgery, during CPB, and at the end of surgery ( °C), hematocrit during CPB (%), duration of body temperature below 32 °C during CPB (min), norepinephrine-equivalent dose (NEE) from the end of CPB to the end of surgery, calculated as NEE = norepinephrine + epinephrine + dopamine/100 + methoxamine/8 + vasopressin×2.5 + ephedrine/10, in µg/kg (35).

### 2.7 Anesthesia management

All patients undergoing general anesthesia for elective cardiovascular surgery must receive a standardized preoperative assessment conducted by a multidisciplinary team. Patients with multiple comorbidities or poor baseline status need an individualized perioperative management plan to optimize preoperative condition, reduce surgical risk, ensure procedural safety, and accelerate recovery. Upon admission, the surgeon performs a comprehensive physical examination and formulates the surgical plan. The surgical team and nurses collect the patient's medical history, including prior diseases, surgical experiences, medications, and lifestyle factors. Preoperative laboratory and imaging examinations are completed to assess overall health, including cardiopulmonary evaluation (e.g., electrocardiogram, pulmonary function tests, echocardiography, coronary angiography) and laboratory tests (e.g., blood counts, biochemical markers, coagulation profile, infection-related biomarkers, liver and renal function, myocardial infarction markers).

All patients fast and abstain from fluids for 10 h preoperatively and receive prophylactic antibiotics at least 40 min before surgery. Patients without cephalosporin allergy receive 2 g cefazolin preoperatively; if the surgery lasts over 3 h, an additional 2 g cefazolin is administered. Cephalosporin-allergic patients may receive 1 g clindamycin intravenously. One hour before surgery, 5 mg subcutaneous morphine is administered for preoperative analgesia. Upon entering the operating room, patients are monitored with five-lead ECG, pulse oximetry, and noninvasive blood pressure. Pure oxygen at 6 L/min is delivered via mask, and an 18G peripheral venous line is established.

After local infiltration with 10 mg lidocaine above the left radial artery, the radial artery is cannulated for invasive arterial blood pressure monitoring. Anesthesia induction is performed with the patient in a head-up ramp position: inhalation of pure oxygen at 6 L/min via mask, followed by slow intravenous injection of 0.4 μg/kg sufentanil citrate. After 3 min, 0.3 mg/kg etomidate is administered. Upon loss of consciousness (MOAA/S score of 0), 0.2 mg/kg cisatracurium is given, and endotracheal intubation is performed when the train-of-four count reaches 0. The tube is secured at the right corner of the mouth, and mechanical ventilation is initiated. Arterial blood gas is measured 5 min after intubation, adjusting tidal volume to maintain PaO_2_ 100–200 mmHg and PCO_2_ 35–45 mmHg based on the P_ET_CO_2_–PaCO_2_ gradient. Anesthesia is maintained with propofol 2 mg/kg·h, dexmedetomidine 0.5 μg/kg·h, cisatracurium 0.15 mg/kg·h, and inhaled 1% sevoflurane. Nasopharyngeal and rectal temperature probes and a urinary catheter are placed. Central venous catheterization is performed in a head-left, Trendelenburg position (<45° tilt) for pressure monitoring.

During skin incision, sternotomy, aortic manipulation such as side-wall clamping or aortic cannulation, CPB initiation, rewarming, hemostasis, and sternal wire closure, sufentanil 0.05–0.1 μg/kg is administered. Arterial pressure is maintained at 80–100 mmHg before arterial cannulation for CPB. Heparin 3 mg/kg is given, and ACT is measured 5 min later; arterial cannulation proceeds when the average ACT ≥360 s, and CPB starts when ACT ≥480 s. After CPB initiation, intravenous fluids are stopped or limited to anesthetic maintenance drugs, and CVP is reduced to near 0 or negative. Ventilation is paused after superior and inferior vena cava clamping, and the expiratory valve is set at 5–10 cmH_2_O for static lung inflation. Rewarming during CPB is performed with vasopressor infusion to achieve effective drug concentration before CPB reduction and reperfusion; low-weight or malnourished patients are rewarmed slowly to nasopharyngeal temperature ≥37.0 °C and rectal or bladder temperature ≥36.5 °C before CPB termination, with active warming to prevent hypothermia-induced coagulopathy.

Before opening the ascending aorta, manual high-tidal-volume ventilation (12–15 mL/kg) is applied in a head-down position to expel air from the heart chambers, coordinated with CPB venous drainage. After vena cava release, mechanical ventilation is resumed to match pulmonary blood flow, and tidal volume and respiratory rate are gradually increased before CPB termination based on P_ET_CO_2_.

After CPB, mechanical ventilation is reassessed, CPB fluid balance and pharmacologic interventions (e.g., furosemide, amiodarone) are reviewed, and protamine is administered to neutralize residual heparin (protamine: heparin ratio 1.5–2.5:1) while monitoring airway pressure, blood pressure, heart rate, CVP, and cardiac function. ACT and arterial blood gas are measured 3 min after protamine completion to guide additional dosing. The FiO₂ is adjusted based on the oxygenation index to maintain PaO₂ at 100–200 mmHg.

At the end of surgery, the anesthesiologist, surgeon, and circulating nurse transfer the patient to the cardiac ICU, providing detailed handover regarding intraoperative events, medication usage, pump-drug concentrations, unresolved issues, and total fluid balance, with documentation and signatures.

### 2.8 Outcomes

#### 2.8.1 Primary outcome

The 30-day follow-up window was selected to encompass the main perioperative recovery period, during which most clinically apparent infections related to the index surgery are expected to be identified, while preserving temporal proximity to the transient intraoperative lactate exposure. With longer follow-up, infections may be increasingly influenced by post-discharge exposures, rehospitalization, subsequent invasive procedures, and additional treatments, thereby weakening their relationship with TWA lactate exposure. Although the specific mechanisms differ, all these infections share common pathways of immunosuppression and impaired host defense. The types of postoperative infections include sepsis, pulmonary infection, surgical site infection, mediastinitis, and infective endocarditis. The postoperative follow-up time is shown in Table 1.

#### 2.8.2 Secondary outcomes

Each component of the composite postoperative infection outcome will be analyzed separately as an exploratory secondary outcome, including sepsis, pulmonary infection, surgical site infection, mediastinitis, and infective endocarditis.

Postoperative 24-hour SIRS score: SIRS refers to the systemic inflammatory response syndrome caused by any pathogenic factor acting on the body. SIRS is diagnosed when two or more of the following criteria are met: (1) body temperature >38 °C or <36 °C; (2) heart rate >90 beats/min; (3) respiratory rate >20 breaths/min or PaCO₂ < 32 mmHg; (4) white blood cell count >12 × 10⁹/L or <4 × 10⁹/L, or immature granulocytes >10%.

Postoperative 24-hour lactate clearance: it will be calculated as follows: [(lactate at the end of surgery−lactate at 24 h after surgery)/lactate at the end of surgery] × 100%.

Mortality: patients who die within 30 days, 3 months, 6 months, and 1 year postoperatively.

Acute kidney injury (AKI): defined according to KDIGO AKI criteria (36): an increase in serum creatinine ≥0.3 mg/dL (≥26.5 μmol/L) within 48 h postoperatively, or an increase to 1.5 times the baseline within 7 days after surgery. In this study, AKI diagnosis relies entirely on elevated creatinine levels. Postoperative sCr peak is defined as the highest recorded creatinine level within 7 days after surgery (37).

Liver injury: postoperative liver injury is defined as elevation of liver enzymes ≥3 times and hyperbilirubinemia (>3 mg/dL or 51 μmol/L) within 3 days after surgery (38, 39).

ICU length of stay: defined as the time from admission to the cardiovascular ICU postoperatively until transfer to the general cardiovascular ward.

Mechanical ventilation duration: defined as the time from admission to the cardiovascular ICU after surgery until extubation in the ICU when extubation criteria are met.

### 2.9 Statistical analysis

Data with a normal distribution will be expressed as mean ± standard deviation (SD), non-normally distributed data as median and interquartile range (IQR), and categorical variables as frequency (n) and percentage (%). Baseline data will be compared using two-sample t-tests, Mann–Whitney U tests, or chi-square tests. Univariate logistic regression analysis will be used to evaluate the association between TWA lactate, covariates, and postoperative infection, calculating odds ratios and 95% confidence intervals (95% CI). The following are the primary analyses: according to the DAG (Figure 4), confounders that are common causes of both TWA lactate and postoperative infection will be adjusted for, whereas mediators and colliders on the causal pathway will not be adjusted. In the model, TWA lactate will be analyzed as both a categorical variable and a continuous variable. Multivariable logistic regression will be employed to assess the risk association between TWA lactate and postoperative infection, reporting adjusted odds ratios (aOR) and 95% CI. For the continuous analysis, aOR will be reported per 0.5 mmol/L increase, per 1.0 mmol/L increase, and per 1-standard deviation increase in TWA lactate. For the categorical analysis, TWA lactate will be dichotomized according to the prespecified cutoff of 2.0 mmol/L. The median of tertiles will be used as a quasi-continuous variable to analyze the linear trend between TWA lactate and postoperative infection risk. Restricted cubic spline regression will be applied to explore the potential nonlinear relationship between TWA lactate and postoperative infection. Threshold analysis will be performed using segmented regression, and the likelihood ratio test will be applied to compare the goodness-of-fit between linear and nonlinear models to identify the optimal model. Based on the determined threshold, an adjusted segmented linear regression model will be constructed to estimate aOR and 95% CI before and after the threshold. The following are the secondary analyses: prespecified subgroup analyses will be conducted according to key characteristics, including age, sex, preoperative comorbidities, and surgery type, and interaction effects will be evaluated. To assess robustness, we will perform sensitivity analyses using lactate load and peak lactate during CPB, and repeat the statistical analyses. To control the error of type I due to multiple comparisons, *P* values for secondary outcomes and prespecified subgroup interaction tests will be adjusted using the Holm–Bonferroni method. All statistical tests are two-sided, and *P* < 0.05 is considered statistically significant.

### 2.10 Handling of missing data

Missing data will be traced and verified, and if they cannot be retrieved, they will be marked as missing in the database. Participants with missing data on the exposure or the primary outcome will not be imputed and will be excluded from the analysis. The proportion and pattern of missing data will be summarized for all variables, including baseline characteristics, covariates, and secondary outcomes.

The missing-data mechanism will be assessed using Little's missing completely at random (MCAR) test. When the data are compatible with MCAR and the variable-specific missingness is below 5%, missing values will be imputed using the k-nearest neighbors (KNN) algorithm. Otherwise, missing values in the minimal adjustment set identified by the DAG and in secondary outcomes will be assumed to be missing at random (MAR) and handled using multiple imputation. The imputation model will include the exposure variable, primary outcome, secondary outcomes, the minimal adjustment set identified by the DAG, and relevant auxiliary variables when available. Multiple imputation will be performed using 20 imputed datasets with a maximum of 50 iterations. Predictive mean matching will be used for continuous variables, while logistic regression will be used for categorical variables. Estimates from the imputed datasets will be combined using Rubin's rules.

As a sensitivity analysis, the primary analysis will be repeated using complete-case data and compared with the results based on multiple imputation. If substantial differences are observed between the two approaches, the potential influence of missing not at random (MNAR) mechanisms on the study findings will be discussed.

### 2.11 Data management

#### 2.11.1 Data collection

All enrolled participants will have their Case Report Forms (CRF) completed by trained research personnel. Data collection will follow a dual-track system, using both paper CRFs and electronic data entry, with subsequent cross-checking for consistency. Face-to-face follow-ups will be conducted for patients who remain hospitalized, while telephone follow-ups will be used for those who have been discharged.

Demographics and baseline characteristics: age (years), sex (male/female), BMI (kg/m^2^), smoking history (yes/no), alcohol consumption (yes/no), and NYHA functional classification (I–IV).

Preoperative comorbidities: hypertension (yes/no), diabetes (yes/no), arrhythmia (yes/no), history of cerebrovascular disease (yes/no), pulmonary comorbidities (yes/no), obesity (yes/no), anemia (yes/no).

Preoperative laboratory and functional indices: preoperative lactate (mmol/L), preoperative blood glucose (mmol/L), hemoglobin (g/L), platelet count (10⁹/L), hypoproteinemia (yes/no), serum total protein (g/L), serum albumin (g/L), lymphocyte count (10⁹/L), monocyte count (10⁹/L), neutrophil count (10⁹/L), TBIL (μmol/L), creatinine (μmol/L), BNP (pg/mL), CRP (mg/L), LvEF (%), cardiac troponin I (CTnI, μg/L), myoglobin (Mb, μg/L), ALT (U/L), AST (U/L), and BUN (mmol/L).

Surgery-related variables: type of surgery (coronary artery bypass grafting, valve surgery, major vascular surgery, cardiac tumor resection, combined surgery, other), surgical time (min), CPB time (min), aortic cross-clamp time (min), intraoperative temperature ( °C), lactate level at the end of CPB (mmol/L), body temperature before surgery, during CPB, and at the end of surgery ( °C); duration of body temperature below 32 °C during CPB (min).

Intraoperative physiological and anesthesia-related parameters: anesthesia duration (min), blood gas indices including lactate (mmol/L), pH, hematocrit (%), blood glucose (mmol/L) and NEE from the end of CPB to the end of surgery (µg/kg).

Postoperative data: Postoperative 24 h: arterial blood gas analysis, hemoglobin (g/L), platelet count (10⁹/L), lymphocyte count (10⁹/L), neutrophil count (10⁹/L), 24-hour lactate clearance (%), 24-hour SIRS score, postoperative complications, PCT (ng/mL), CRP (mg/L). Duration of mechanical ventilation (hours), ICU length of stay (hours). Creatinine (μmol/L) within 7 days postoperatively, and alanine aminotransferase (ALT, U/L) and aspartate aminotransferase (AST, U/L) levels within 3 days postoperatively.

#### 2.11.2 Data entry

All study data will be entered into the research database independently by two trained personnel. After entry, the system will automatically generate a logic-check report to identify out-of-range values, missing data, or logical inconsistencies. Any discrepancies will be verified and corrected by researchers against the original medical records or test reports.

#### 2.11.3 Data storage and security

The database will implement multi-level access control in FREE Electronic Data Capture System (Beijing Free Clinical Medical Technology Co., Ltd, Beijing, China): only the principal investigator and data administrator will have full access, while other study personnel will access data according to their permissions. All electronic data will be stored on an encrypted server with regular backups. Paper CRFs will be securely stored in a locked cabinet. Personally identifiable information (e.g., name, hospital ID) will be converted to a unique study code upon entry to ensure privacy.

#### 2.11.4 Data quality control

All study personnel will receive standardized training before study initiation, covering data collection methods, standard definitions, and form-filling procedures. Regular onsite or remote monitoring will be conducted, with random case checks against original medical records to verify accuracy. Key variables (e.g., lactate levels, postoperative infection diagnoses) will undergo dual verification by two independent researchers.

#### 2.11.5 Data lock and export

Once data cleaning and verification are complete, the principal investigator and data administrator will jointly confirm the dataset for locking. The locked dataset will serve as the official analysis database and must not be modified arbitrarily. Any required revisions must be documented and fully traceable. Statistical analysts will use only the locked, anonymized dataset for analysis.

#### 2.11.6 Confidentiality and retention

All study data, CRFs, and monitoring records will be retained for at least 5 years after study completion. Any publication or data sharing requires ethical committee approval and must be anonymized before use.

## 3 Discussion

Postoperative infection after cardiac surgery with CPB is a common and serious complication, significantly increasing hospital stay, reoperation rates, and mortality (40, 41). Although prior studies have shown that lactate levels are associated with various adverse postoperative outcomes (42–44), research specifically examining the relationship between TWA lactate levels during CPB and multiple types of postoperative infection remains limited. This study aims to fill this gap. Compared with previous studies, the present study has notable innovations: lactate levels are assessed using TWA during CPB, a dynamic indicator that accounts for changes over time and better reflects overall lactate burden (45); in addition, various types of postoperative infections are clearly defined as outcomes, providing a more comprehensive and rigorous assessment.

This prospective cohort study will systematically record preoperative and intraoperative information at patient enrollment and follow up on postoperative infection events, ensuring data completeness and temporal consistency, which improves inference. Strict definitions for exposure and outcomes are used, with infection diagnoses based on internationally accepted standards and independently verified by study personnel and attending physicians, ensuring study rigor. Comprehensive covariates—including demographics, laboratory tests, and surgical factors—will be collected to minimize confounding and more reliably evaluate the independent association between lactate and infection. Additionally, this study incorporates a DAG to guide covariate selection and clarify the assumed causal structure between intraoperative lactate exposure and postoperative infection, thereby enhancing the transparency and reproducibility of covariate selection and model specification.

Our study has several limitations. This is a single-center cohort study conducted at a large cardiovascular center. Variations in CPB management protocols and perioperative care practices may differ from those at other institutions. These differences could influence lactate levels as well as clinical outcomes, thereby limiting the generalizability of the study findings. Lactate is a dynamic variable, and using TWA during CPB may miss some peak values despite capturing overall exposure. Additionally, combining different outcomes may obscure differential associations between lactate and specific infection types. For example, lactate may have a stronger association with pneumonia (due to its effects on pulmonary microcirculation) and a weaker association with mediastinitis or wound infections (which are primarily related to surgical technique and dressing change frequency). Moreover, the 30-day follow-up window may not capture infections with delayed onset, particularly infective endocarditis, and may therefore underestimate the overall postoperative infectious burden. Accordingly, the findings of this study will primarily reflect the association between intraoperative TWA lactate and early postoperative infection.

If TWA lactate during CPB is found to be significantly associated with postoperative infection risk, it could serve as a potential early risk-stratification and intervention tool. Lactate monitoring is simple and cost-effective; elevated levels may prompt enhanced postoperative surveillance, optimized intraoperative management, and immunonutrition support, enabling early identification and intervention for high-risk patients. Future studies should consider large-scale, multicenter cohorts. Basic research should explore the biological mechanisms linking lactate levels and postoperative infection, including specific pathways of immune suppression and inflammatory responses. Finally, interventional studies should focus on methods to reduce lactate levels after CPB and whether lowering lactate can decrease postoperative infection rates, providing stronger evidence for clinical practice. This study aims to offer new biomarker combinations and decision-support tools for early warning and intervention of postoperative infection in cardiac surgery, ultimately improving patient outcomes and reducing healthcare costs.

## Data Availability

The datasets for this study will be saved in the Data Management Platform of The Affiliated Hospital of Qingdao University (https://www.clinicaledc.com). To obtain access to these data, please reach out to the corresponding author.
